# The use of midurethral sling in the correction of low-stage cystoceles: a prospective randomized trial

**DOI:** 10.1007/s00192-023-05691-2

**Published:** 2023-12-04

**Authors:** Diaeldin T. Ramadan, Ahmed S. Elhefnawy, Bassem S. Wadie

**Affiliations:** Urology and Nephrology Center, Mansoura University, Gomhorria Street, Mansoura, 35516 Egypt

**Keywords:** Incontinence, Cystocele, Slings, Colporrhaphy, Comparison

## Abstract

**Introduction and hypothesis:**

The objective was to assess whether midurethral slings (MUS) can improve both stress urinary incontinence (SUI) and cystoceles. MUS with anterior colporrhaphy (AC) as a treatment for SUI with cystocele is more invasive and carries greater risk than MUS alone.

**Methods:**

This is a prospective randomized study involving women with stage 1 or 2 cystocele and SUI, who were > 21 years of age, who had had no previous surgery for SUI. Predominant SUI, symptomatic anterior pelvic organ prolapse, and informed consent were mandatory. Patients were randomized as to whether AC had been performed. The sling procedure was left to the surgeon’s discretion: pubovaginal sling, tension-free vaginal tape, or trans-obturator tape. Success was defined as a negative stress test and no evidence of cystocele upon local examination.

**Results:**

Ninety-eight patients were enrolled, 48 underwent MUS, and 50 underwent MUS and AC. Mean age ± SD was 44.96 ± 8.13 years. Baseline characteristics were similar. Operative time and blood loss were significantly higher in the MUS/AC group (*p* = 0.01 and 0.02 respectively). At 3 months, success was 79.1% and 77.8% in the MUS and MUS/AC groups respectively. This was maintained until 6 months (79.1% and 77.8% respectively). At 1 year, the results were comparable with success rates of 96.2% and 87.0% in the MUS and MUS and AC groups respectively. Symptom scores were comparable at 6- and 12-month evaluations.

**Conclusion:**

Midurethral slings correct symptomatic stage 1 or 2 cystoceles without the need for AC, which carries the risk of a significantly longer procedure and more significant blood loss.

## Introduction

Midurethral sling (MUS) is the standard treatment for stress urinary incontinence (SUI). The association of SUI and anterior pelvic organ prolapse (POP) is common. It is suggested that 40–60% of women with SUI might require a concurrent procedure for POP at the time of surgery [1]. In a previous study, we reported 32 women with anterior POP (8 had stage 1 and 24 stage 2) among 53 women with SUI who were planning to undergo surgery [2].

It was previously noted that MUS alone could correct POP at a mean follow-up of 62.6 months [3], where an autologous pubovaginal sling (PVS) seems to be a safe and effective correction of cystocele with or without SUI in 30 patients with symptomatic cystocele, including 14 with and 16 without concomitant SUI. However, the urological literature provides scarce data on whether a sling alone can cure symptomatic low-stage cystoceles.

Our study is aimed at showing that a sling is a treatment for low-stage cystocele-associated SUI.

## Patients and methods

This is a prospective randomized trial. Patients underwent a stress test that is positive if leakage was observed while the patient coughed in the lithotomy position with the bladder 200 ml full. POP was staged according to the POP Quantification (POPQ) [4]. A brief neurourological examination was conducted, including perineal sensation, knee, ankle jerks, and tone of the anal sphincter. Only those with stages 1 or 2 anterior POP were enrolled if they were older than 21 years, had had no previous surgical intervention for SUI, had had predominant stress incontinence, had had symptomatic anterior POP, and had provided informed consent. Women with stages > 2 anterior POP were excluded.

Urodynamic testing was performed and included filling and voiding cystometry. Medium fill water cystometry (50 ml/min) using a dual lumen 8 Fr catheter was performed. The technique, definitions, and units conformed to the standards proposed by the International Continence Society [5]. Compliance was calculated at a maximum cystometric capacity/detrusor pressure at maximum capacity.

Stress incontinence was defined as involuntary leakage upon effort or exertion [5]. Surgery was performed by one of two surgeons. Each had a track record of at least 100 cases of MUS. Objective success was defined as no evidence of stress incontinence on the stress test, a negative pad test, and no evidence of anterior POP upon clinical examination.

Written informed consent was obtained from the women enrolled, and IRB approval was obtained from the urology department (Approval # 2017_009).

Patients were randomized using closed envelopes. The type of anti-incontinence procedure was left to the surgeon’s discretion. For PVS, a technique similar to that previously described [2] was used with some modifications. The sling was shorter (6–8 cm) and was fixed to the underlying periurethral fascia using 4/0 polyglactin sutures at the 6 and 12 o’clock positions. Tension-free vaginal tape (TVT) was performed in accordance with the technique postulated by Ulmsten et al. [6], with retrograde passage of the trocar and localized incisions over the tip of the trocar and check cystoscopy. Transobturator tape (TOT) was carried out in accordance with the description by Delorme [7], with the trocar passing from inside-out. In those who were randomized to receive anterior colporrhaphy (AC), a standard procedure was adopted. AC was performed using three sutures of 2/0 polydioxanone, approximating the pubocervical fascia in the midline.

The primary outcome was the cure of POP as indicated by local examination. The secondary outcome measure was cure of incontinence as indicated by negative stress and 1-h pad tests. Treatment failure was defined as recurrence of stress incontinence, as demonstrated by the positive stress test or positive pad test (stress failure) or recurrence of the same stage of prolapse upon local examination.

Statistical analysis was conducted using SPSS version 22. Continuous normal data were compared using Student’s *t* test. Independent samples *t* tests were used to study the difference between the means. The Chi-squared test was used for nonparametric data. The *p* value was considered significant if < 0.05.

The statistical power of the study was calculated considering the success rate of 50% in any arm, an alpha error of 0.05 with a 1:1 enrollment ratio and a power of 80%. The calculated sample size was 116 and the margin of error was 5.94% (there was a 80% chance that the real value was within ± 5.94% of the measured value).

**Fig. 1:**
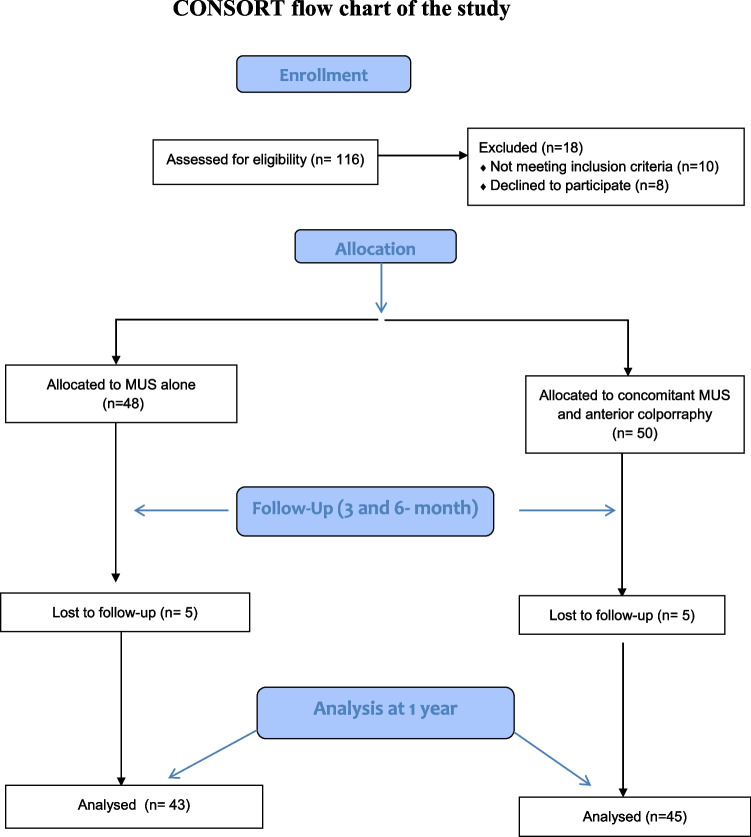
Consolidated Standards of Reporting Trials flow chart of the study

Figure 1 demonstrates the Consolidated Standards of Reporting Trials flow chart of the study.

## Results

A total of 98 patients with a diagnosis of SUI and anterior POP were enrolled in this study, after exclusion of 10 patients with associated bladder/urethral pathology and 8 who withdrew consent and refused to enroll in the study after admission. Forty patients underwent PVS, 38 TVT, and only 20 underwent TOT. Forty-eight patients underwent MUS alone (group 1), whereas 50 underwent MUS/AC (group 2). Most patients completed the 12-month follow-up.

The mean age was 44.96 ± 8.13 years (range 42 to 60). All women had SUI and vaginal bulge as the primary complaints. A history of abdominal hysterectomy was observed in 7 patients, whereas 6 had had AC. The baseline clinical variables are illustrated in Table 1.

**Table 1:** Patients’ characteristics

		MUS (*n* = 48)	MUS and AC (*n* = 50)	*p* value*
Age (years)		44.96 ± 8.13	47.05 ± 8.12	0.2
Gravidity		3.77 ± 1.49	4.03 ± 1.51	0.4
Parity		3.28 ± 1.21	3.44 ± 1.10	0.5
BMI		31.83 ± 5.09	32.58 ± 4.69	0.4
1-h pad test		25.34 (20–115)	25.59 (20–125)	0.56**
POP grade 1		14	16	0.6
Grade 2		34	34	0.6
HB (g/dl)		12.52 ± 0.92	12.29 ± 1.22	0.3
ASA#	I	33 (68.75%)	27 (54%)	0.4
	II	13 (27.05%)	16 (32%)	
	III	2 (4.20%)	7 (14%)	
Sling type	TVT	15 (31.25%)	19 (38%)	0.34
	TOT	18 (37.50%)	17 (34%)	
	PVS	15 (31.25%)	14 (28%)	
Mean operative time (min) ± SD		44.67 ± 19.95	55.93 ± 21.99	**0.01**
Mean blood loss (ml) ± SD		45.00 ± 21.40	67.02 ± 23.71	**0.02**
Mean PVR (after catheter removal)		11.7 ± 38.8	8.26 ± 20.82	0.59

Significant *p* values are shown in bold

*AC* anterior colporrhaphy, *ASA#* American Society of Anesthesiologists physical status classification, *BMI* body mass index, *HB* hemoglobin, *POP* pelvic organ prolapse, *PVR* postvoid residual, *PVS* pubovaginal sling, *TOT* transobturator tape, *TVT* tension-free vaginal tape

*Independent *t* test

**Mann–Whitney test

In the first group (MUS only; *n* = 48), the mean postvoid residual was 10.08 versus 10.86 in group 2 (MUS/AC; *n* = 50; *p* = 0.70), and the mean Matthews correlation coefficient (MCC) was 403.47 ± 98.04 vs 379.02 ± 111.25. The mean compliance was 50.00 ± 19.79 in group 1 (*p* = 0.29) and 62.81 ± 18.64 in group 2 (*p* = 0.25). Detrusor overactivity (DO) was noted in 3 patients in group 1 (6.25%) and in 4 (8%) in group 2 (*p* = 0.59). The mean Valsalva leak-point pressure was 82.75 ± 33.5 cmH_2_O in group 1, whereas it was 79.14 ± 28.33 (*p* = 0.61) in group 2. The mean maximum flow rate (Qmax) was 22.80 ± 10.99 ml/s in group 1 and 23 ± 11.06 ml/s in group 2 (*p* = 0.87). The mean detrusor pressure Qmax was 23.95 ± 21.26 in group 1 and 16.96 ± 8.26 cmH_2_O in group 2 (*p* = 0.09) using an independent *t* test. Table 2 demonstrates the baseline urodynamics.

**Table 2:** Baseline urodynamics parameters

	MUS (*n* = 48)	MUS and AC (*n* = 50)	*p* value*
Mean PVR ± SD (ml)	10.08 (0–40)	10.86 ± (0–80)	0.70
Mean MCC ± SD (ml)	403.47 ± 98.04	379.02 ± 111.25	0.29
Mean compliance ± SD (ml/cmH_2_O)	50.00 ± 19.79	62.81 ± 18.64	0.25
DO (%)	3 (6.25%)	4 (8%)	0.59
Mean VLPP ± SD (cmH_2_O)	82.75 ± 33.50	79.14 ± 28.33	0.61
Mean Qmax ± SD (ml/s)	22.80 ± 10.99	23.21 ± 11.06	0.87
Mean Pdet Qmax ± SD (cmH_2_O)	23.95 ± 21.26	16.96 ± 8.26	0.09

*AC* anterior colporrhaphy, *DO* detrusor overactivity, *MUS* midurethral sling, *Pdet* detrusor pressure, *PVR* postvoid residual, *Qmax* maximum flow rate, *VLPP* Valsalva leak-point pressure

*Independent *t* test

As shown in Table 1, the mean operative time and volume of blood loss were significantly higher in the colporrhaphy group than in the MUS alone group (*p* = 0.01 and 0.02 respectively).

At 3 months, local PV examination was comparable between the groups, with no evidence of POP in 77.1% and 76% of the MUS and MUS/AC groups respectively. Only one case in the MUS/AC group had vaginal extrusion, and she was managed using tape excision. One case from the MUS group had recurrent incontinence (positive stress test/pad test > 2 g). One case in the MUS/AC group had recurrence of SUI, 1 had recurrence of the POP, and 1 had recurrence of both incontinence and prolapse. No significant difference was detected between the groups regarding PVR, pad test, and flow parameters.

Three failures were managed by redo AC, redo MUS, and combined MUS/AC.

At 6 months, no significant difference between the groups regarding PVR, pad test, and flow parameters was noted. De novo DO developed in 8 patients (18.6%) from the MUS group and in 5 patients (11.1%) from the MUS/AC group.

At 1 year, there were 43 from the first group and 45 from the second group who had completed evaluation. No more failures were reported by those who were available to the last follow-up. Tables 3, 4, and 5 demonstrate outcome measures at different follow-up intervals. The differences in Urogenital Distress Inventory-6 and Incontinence Impact Questionnaire-7 scores at 6 and 12 months were insignificant, as shown in Table 6.

**Table 3:** Outcome at 3 months

			MUS (*n* = 48)	MUS/AC (*n* = 50)	*p* value
Local examination		Normal	37 (77.1%)	38 (76%)	0.72**
		Grade I	9 (18.75%)	9 (18%)	
		Grade II	2 (4.15%)	3 (6%)	
Complications		Extrusion	0 (0%)	1 (2%)	0.45**
Treatment failure		MUS failure	1 (2.3%)	1 (2%)	
		AC failure	0 (0%)	1 (2%)	
		Both	0 (0%)	1 (2%)	
Pad weight gain (g) mean ± SD			4.57 ± 17.85	5.26 ± 16.14	0.85*
Qmax (ml/s) mean ± SD			27.41 ± 12.47	26.37 ± 13.96	0.72*
PVR (ml) mean ± SD			18.73 ± 37.51	8.02 ± 14.69	0.10*
Pus cells in urinalysis			8 (16.66%)	5 (10%)	0.27**

*AC* anterior colporrhaphy, *MUS* midurethral sling, *PVR* postvoid residual, *Qmax* maximum flow rate

*Independent *t* test

^**^Chi-squared test

**Table 4:** Outcome at 6 months

			MUS (*N* = 48)	MUS/AC (*N* = 50)	*p* value
Local examination	Normal		37 (77.1%)	38 (76%)	0.22**
	Grade I		9 (18.75%)	9 (18%)	
	Grade II		2 (4.15%)	3 (6%)	
Complications (new onset)			0	0	
Pad test, g (mean ± SD)			4.9 ± 15.66	5.8 ± 14.3	0.73*
Pus cells in urinalysis			6 (13.95%)	7 (15.5%)	0.42*
Qmax ml/s (mean ± SD)			23.50 ± 13.22	27.18 ± 17.67	0.72*
PVR ml (mean ± SD)			1.37 ± 6.69	2.70 ± 16.43	0.10*
MCC ml (mean ± SD)			370.2 ± 108.46	352.51 ± 105.49	0.52*
Compliance ml/cmH_2_O (mean ± SD)			41.76 ± 27.00	47.71 ± 41.03	0.51*
De novo DO			8 (16.6%)	5 (10%)	NA
Pdet Qmax cmH_2_O (mean ± SD)			23.50 ± 13.22	27.18 ± 17.67	0.42*

*AC* anterior colporrhaphy, *DO* detrusor overactivity, *MCC* Matthews correlation coefficient, *MUS* midurethral sling, *NA* not available, *Pdet* detrusor pressure, *PVR* postvoid residual, *Qmax* maximum flow rate

*Independent *t* test

^**^Chi-squared test

**Table 5:** Outcome at 1 year

			MUS (*N* = 43)	MUS/AC (*N* = 45)	*p* value
Local examination	Normal		36 (83.8%)	37 (82.22%)	0.42**
	Grade I		4 (9.3%)	6 (13.33%)	
	Grade II		3 (6.9%)	2 (4.45%)	
Pad test, g (mean ± SD)			2.59 ± 6.068	9.20 ± 31.645	0.27*
Qmax ml/s (mean ± SD)			25.81 ± 7.69	25.87 ± 8.98	0.98*
PVR ml (mean ± SD)			6.33 ± 34.50	1.44 ± 4.21	0.82*
Pyuria in urinalysis			4 (15.4%)	1 (4.3%)	0.35*

*AC* anterior colporrhaphy, *MUS* midurethral sling, *PVR* postvoid residual, *Qmax* maximum flow rate

*Independent *t* test

^**^Chi-squared test

**Table 6:** Comparison of Incontinence Impact Questionnaire-7 (*IIQ-7*) and Urogenital Distress Inventory-6 (*UDI-6*) scores at 6 months and 1 year

	MUS	MUS and AC	*p* value*
IIQ-7 at 6 months (mean ± SD)	6.55 ± 4.90	6.37 ± 4.09	0.87
UDI-6 at 6 months (mean ± SD)	3.94 ± 5.24	4.59 ± 5.83	0.64
IIQ-7 at 1 year (mean ± SD)	4.47 ± 5.59	5.50 ± 6.85	0.8
UDI-6 at 1 year (mean ± SD)	7.00 ± 4.61	7.20 ± 4.31	0.5

*AC* anterior colporrhaphy, *MUS* midurethral sling

*Paired sample *t* test

## Discussion

Our results showed that MUS can cure low-stage anterior POP. Previous reports suggested that PVS might provide additional support to the bladder base, improving the durability of AC [8]. Colombo et al. [9] conducted a trial comparing Burch colposuspension (35 cases) with AC (22 cases) in the treatment of SUI concomitant with stage 2/3 cystocele. They concluded that neither is an effective treatment. Another trial by Kammerer-Doak et al. [10] found that Burch colposuspension was significantly better than modified AC in the correction of SUI in a randomized trial.

Going through different POP grading systems, Muir et al. [11] found that Baden Walker was used in 19.8% of the 146 articles they studied, next only to the POPQ, which was reported by 22.6% of studies. Correction of anterior POP is traditionally achieved by AC, a technique that has undergone few modifications over time [12]. Weber and Walters [13] concluded that the tissue plicated during AC is probably the vaginal muscularis, not a true fascial layer.

Anterior colporrhaphy was thought to be curative in SUI in the study by Tamussino et al. [14], where AC cured 61% of women (65 out of 107) of their incontinence at 5 years. However, many studies refute this conclusion. Hutchings and Black [15] studied 221 women with SUI and found that the cure rate varied by procedure (colposuspension 34% dry; needle suspensions 13%; AC 19%). After adjusting for confounders, colposuspension was significantly more likely to result in an improvement than AC. Furthermore, Khayyami et al. [16] concluded that AC was associated with a decrease in abdominal pressure of 50 cmH_2_O (P_O-Abd 50_) at a median 2-year postoperative follow-up in 28 women with this procedure performed as a treatment for cystoceles. They concluded that the urethral closure mechanism deteriorated after AC.

Although the use of meshes for the correction of cystocele was favorably considered by many [17–20], the FDA warning in 2011 resulted in a drastic decline in the use of mesh in correction of cystoceles and SUI [21].

Pubovaginal sling using the rectus sheath was found to be an effective treatment for symptomatic cystoceles in 30 women [3]. However, the sling described was a large trapezoid graft that is fixed by four sutures rather than two. Our results show that standard MUS can cure a concomitant stage 1 or 2 cystocele, and the difference between the two groups was insignificant at all follow-up points, considering the recurrence of cystocele or the cure of SUI as evidenced by the stress and pad tests. We also found that regardless of the type of sling used, the impact on concomitant stage 1 or 2 cystoceles was the same.

The difference between the two groups regarding symptoms was insignificant at 6 and 12 months. This means that concomitant colporrhaphy did not affect the patients’ perception of their quality of life.

It was debated that repair of stage 2 cystoceles could be omitted altogether from surgery for SUI associated with cystoceles [22]. Park et al. studied 92 women with SUI and asymptomatic stage II cystocele who were divided into a TVT and concomitant cystocele repair group and found no difference in the surgical outcome and lower urinary tract symptoms between the TVT sling-only group and the concomitant repair group [22]. Our study has shortcomings: the sample size is small; the application of three different sling techniques would confound the outcome of the sling surgery. However, based on the study by Jeon et al. [23], PVS and TVT seem to be more efficacious than TOT at 2 years. Our patients’ cohort is homogenous regarding the age, parity, BMI, and the severity of incontinence at baseline, which is a considerable strength. Nevertheless, all patients were recruited from single-center OPD, which could compromise the generalization of our results. Longer-term follow-up is preferable, considering the natural history of the sling and AC surgeries and a follow-up of a minimum of 5 years is desirable before drawing a firm conclusion. Besides, the best-case scenario would have been the blinding of the investigators as to outcome during the follow-up period.

In conclusion, MUS can correct symptomatic stage 1 or 2 cystoceles without the need for added AC. Adding colporrhaphy was associated with a significantly longer procedure and greater blood loss. Although three different sling techniques were adopted, which is potentially confounding to the outcome of the stud, this could be taken as a strength, as we utilized the three most popular MUS in the same study.

## Data Availability

Data are available upon request to the corresponding author.

## Abbreviations

AC: Anterior colporrhaphy
DO: Detrusor overactivity
HB: Hemoglobin
IIQ-7: Incontinence Impact Questionnaire-7
MCC: Matthews correlation coefficient
MUS: Midurethral sling
POP: Pelvic organ prolapse
POPQ: Pelvic Organ Prolapse Quantification
PVR: Postvoid residual
PVS: Pubovaginal sling
Qmax: Maximum flow rate
SUI: Stress urinary incontinence
TOT: Transobturator tape
TVT: Tension-free vaginal tape
UDI-6: Urogenital Distress Inventory-6
VLPP: Valsalva leak-point pressure
